# Hospital and Institutional News

**Published:** 1920-08-07

**Authors:** 


### HOSPITAL AND INSTITUTIONAL NEWS.

### THE FEDERATION OF MEDICAL SOCIETIES.

On page 481 of this issue we publish the Presidential address delivered by Sir Malcolm Morris at the annual meeting of the above Federation, and would call the attention of all those interested in medical progress and politics to the essentially valuable views which the speaker puts forward. The work which the Federation has already done is too well known to call for further emphasis here, but, as a statement of its future aims and policy, we feel that the Presidential address should be more than closely studied. The great urgency is for further general and professional support towards an organisation which bids fair to provide opportunity for development of that greatly-needed unity of professional opinion at a time when the national aspect of medicine is of the utmost importance.

### THE FUTURE OF THE METROPOLITAN ASYLUMS BOARD.

It will be remembered that late last year a special committee of the London County Council reported on the organisation of the health services of London, and a summary of the proposals of this committee was published in The Hospital of January 10 last (page 336). The scheme—one of redistribution and re-arrangement—naturally affected the constitution and duties of the Metropolitan Asylums Board, and the General Purposes Committee of that body having reported at length on the matter, the Board has accepted the conclusions arrived at. As is well known, the Metropolitan Asylums Board, under its constitution, is not appointed by direct election, but its duties have been generally acknowledged to have been most efficiently carried out. It would be a distinct disadvantage to transfer the whole system of managing the chief infectious hospitals of London and such other important undertakings as the treatment of tuberculosis, etc., direct to another health authority, thus losing the counsel of those who have gained great experience in these matters. It is therefore proposed, as an alternative to transferring *en bloc* the whole organisation, to widen the representation of the public on the Metropolitan Asylums Board by co-opting members of the London County Council and the Metropolitan Borough Councils. It is also proposed by the Board that its name should be changed to one more clearly indicating the nature of the work. These steps, which on the face of them are wise and progressive, are, in the opinion of the Metropolitan Asylums Board, the best policy, pending an authoritative inquiry as proposed by the London County Council into the question of London government as a comprehensive whole.

### SCOTLAND'S SOUND FINANCE.

Sir Napier Burnett, Director of the Hospital Service Department of the British Red Cross, has added an interesting commentary to the report of the survey he has made of the voluntary hospitals of Scotland to ascertain the effect of the war on their finances. The survey covered the years 1915-19 inclusive, and Sir Napier says it shows that the Scottish hospitals, taken as a whole, have not experienced the financial difficulties hospitals in other parts of the kingdom have had to contend with. They were in a sound financial position up to the end of 1918, and received as ordinary income and "free" legacies more than was necessary to meet their ordinary expenditure. The following year, however, was a bad one, and it is estimated that about a quarter of a million pounds will be required to put them back into their pre-war position. "Free" legacies, that is to say such as are not earmarked for any special purpose, Sir Napier found to be a marked feature over the Tweed. Citing the large sums coming in this manner to the big hospitals attached to medical schools in Scotland, he holds this indicated that the Scottish people are maintaining their old love of education, believing that they can show it in no better way than by leaving their money to these institutions.

### EX-SERVICE MEN'S WARD AT CARDIFF.

The Board of Management of King Edward VII. Hospital, Cardiff, after a discussion, of which more in a moment, decided that, as promised on their behalf by Sir William James Thomas to the Cardiff Ex-Service Men's Council, one of the wards shall be named "The Ex-Service Men's Ward," in grateful recognition of the generous gift of £6,000 which the men received from the "Byng Millions" to the Lord Mayor's Hospital Fund. One of the ex-Service men's representatives on the Board said they were also ready to shoulder the responsibility of raising the money for the upkeep of the ward thus called into being. The proposal to carry out the promise to name the ward was opposed, strangely enough, by a few of the members, who asserted that the soldiers were under a moral obligation to give their money to the hospital, because it had cost more than £6,000 to treat the soldiers there during the war. This line of argument was interrupted by cries of dissent from other members. In the end the Board marked their appreciation of the self-sacrificing gift of the soldiers by adhering to the proposed naming of the ward, and the motion received the support of all but two of the members. In regard to the latter, we are tempted to follow the example of the House of Commons and go to the extreme length of naming them.

### ROYAL VICTORIA INFIRMARY, NEWCASTLE.

When the crisis came in 1919, the Subscriptions Committee of the Royal Victoria Infirmary, Newcastle-upon-Tyne, issued to every subscriber and unit of workmen contributors, and to all country district committees, a circular which included an article reprinted from The Hospital. The appeal was followed by interviews, correspondence, and a public meeting. The result in six months has been to add £10,000 to the income for the period, and it is estimated that at least £20,000 additional will be collected this year, apart from special efforts which are being organised to clear off the overdraft at the Bank of £24,000 consequent upon the deficiency for 1919. The organisation of income at this hospital is undertaken by a special committee —the Chairman of which is Mr. Charles Irwin, J.P., and Mr. A. Fletcher, F.C.I.S., the Financial Secretary of the institution. Since the inauguration of the Committee in 1905, £45,000 has been added to the annual income. The inception of the Special Committee was due to Sir Riley Lord, who had organised the fund for building of the new infirmary opened in 1906. Sir Riley, who has attained his eighty-third year, is the present Chairman of the Finance Committee. The Newcastle Infirmary has a fine record in the organisation of workpeople's contributions. The income from this source is about £30,000 a year. The collections are made by voluntary deductions from wages at the collieries, shipyards, factories, etc., on Tyneside and in the counties of Northumberland and Durham.

### THE CHARITY COMMISSIONERS AND ENFIELD COTTAGE HOSPITAL.

THE draft scheme for the future management of Enfield Cottage Hospital has now been issued by the Charity Commissioners. The scheme, which is not in any way exceptional, is chiefly interesting for the means which it proposes to avoid the recurrence of the disputes which led to the inquiry. It provides for a house committee of seven, three of whom at least are to be women, from the committee of management, and one "medical manager" appointed by the medical officers. The medical staff is to consist of local practitioners approved by the managing committee. The scheme, while vesting the right of sending patients in any medical officer or practitioner "residing in and practising near" the district, gives no doctor not a medical officer the right to treat the patient in hospital. The district councillors have criticised the scheme because it has not concerned itself with the negotiations between the Cottage Hospital and the War Memorial Hospital Committee, negotiations which proposed to transfer the Cottage Hospital from the present trustees to the Memorial Committee. Since, however, time is allowed for objections to the scheme of the Charity Commissioners, there does not seem much ground for complaint. The negotiations in question were begun, we believe, after the Commissioners had been compelled to intervene. A portrait of the late Mr. James Penny, who filled the post of honorary secretary for twenty-eight years, has been presented to each of his two daughters, with an album containing the names of the subscribers.

### MAINTENANCE CHARGE DEFEATED AT BIRMINGHAM.

IT is noteworthy that the Governors of Birmingham General Hospital have declined the Board's recommendation to make a maintenance charge of one guinea upon all in-patients able to pay. The proposal, therefore, has been deferred for six months. The opposition came from a representative of the Hospital Saturday Fund and from the delegates sent by the various works, who agreed that, if the proposal was carried, the men would withdraw their weekly contributions. Meantime a hospital league is being formed to gather additional subscriptions, and in six months' time it will be seen how much income can be raised from this source. Supposing the league to fail, it remains to be seen whether the workmen's delegates will take more kindly to the proposal. It is, at all events, clear that, just as the proposal for a maintenance charge came from those hospitals which had no methodical system of revenue, but relied on general personal appeals, so it is even less liked in districts where weekly contributions are well organised. Meantime, we hope that the postponement of the proposal in Birmingham will strengthen the hands of the Saturday Fund and lead to the inclusion of every employer and every firm in its ranks. That is the real solution.

### TAX-FREE BENEFACTIONS.

IT is said that the concession made by the Chancellor of the Exchequer in respect of reduction of excess profits duty by means of gifts to hospitals and institutions has not met with much enthusiasm amongst those interested in the voluntary hospitals, not entirely because of the small extent of the concession, but because the benefit, which will not operate until next year, is so remote that it cannot affect the present financial crisis. In the recent debate, however, Earl Winterton, who is Chairman of the Hospital for Women and a member of the Council of the British Hospitals Association, said that he was deeply grateful to the Chancellor for the concession, which he considered was quite as much as could be expected. As it is not altogether clearly understood what the concession amounts to, it may be helpful to quote the illustration used by one of the speakers in the recent debate in the House of Commons. If a man gives £100,000 out of the profits of his business he will have excess profits duty remitted on £20,000, provided that the £20,000 is not more than five per cent. of the total profits of the year.

### NEW WING OPENED AT MARGATE.

PRINCESS ALICE has now opened the King George the Fifth Wing for discharged service men at the Royal Sea Bathing Hospital, Margate. Archdeacon Holmes conducted the dedication service, and Dr. O. Shah, the resident surgeon, and Miss Kempton, the matron, escorted the Princess through the wards. At present there are nearly 300 patients in the hospital. A bazaar was also opened by the Princess, and at the ceremony Sir Dyce Duckworth and Sir H. Lennard were among the speakers. The new motor-ambulance belonging to the town was on view, with the ambulance corps in attendance.

### THE ALEXANDRA HOSPITAL'S EXPERIMENT.

THE transference of the Alexandra Hospital for Crippled Children to the hills of Caversham from Queen Square, Bloomsbury, to some extent depends upon the success of the hospital's appeal, which is remarkable because it is only the second issue since it was founded in 1867. The Caversham site has been bought, but the scheme needs £20,000 quickly, and £50,000 in all. Towards the immediate sum a conditional gift of £3,000 has been promised. The hospital has had for nearly twenty years a country branch at Clandon, and its patients the use of twelve beds at a convalescent home at Painswick, in Gloucestershire. But these advantages are very different from that proposed by setting the hospital itself in the country. It remains, we hope, to be proved that this unusual resort to an appeal will have a response no less out of the ordinary.

### A GREEK GIFT.

The presentation of an expensive apparatus is not usually made an occasion for the drastic criticism of a hospital, but the home-truths to which the Management Committee of the Guest Hospital, Dudley, has long turned a deaf ear were again repeated when a new operating table was formally bestowed on the hospital at a recent fête. Sir George Bean, who presented the gift, which the Dudley War Pensions Committee secured by public subscription, declared that the management was stereotyped, and that the working classes should share in it. The representative of the Workmen's Saturday Committee, Mr. F. J. Ballard, used stronger language, and described the committee as a group of "old fossils." At the annual meeting in November some of these would be replaced by representatives of the working classes, whose Saturday Committee was busy organising weekly contributions. The hospital was likely to be £12,000 in debt. The thanks of the committee are not recorded, but surely the members must realise at last that, however complacent they may be over their own efforts, they are out of sympathy with the public, which will soon tire of sugaring its criticism with presents. We hope that the annual meeting will see a change, and that all supporters who are also critics will attend and elect a more representative committee.

### MR. WORMELL'S EXPERIENCE.

The organisation of sectional subscriptions in Coventry on behalf of the Coventry and Warwickshire Hospital is making progress, and a different scheme is formed for different classes. The manufacturers have already responded, we do not know how generally this section has been covered, and there is another scheme for retailers, of which the results will soon be known. Stating these facts, Mr. W. J. Wormell said that the movement in support of the hospital seemed to be spreading; and indeed, one of the advantages of the plan is that once started, class after class tends to organise support in the same way. It is curious, while workmen's subscriptions have multiplied in every centre where they have been started, that it has taken so long to form, or even persuade people to attempt to form, employers' subscription funds. The retailer is still generally neglected, yet retail shops are the industry of town after town where there are few or no factories. We hope that Mr. Wormell's experience will be pondered elsewhere, and lead to kindred attempts. The scope is immense, and its methodical development is unaccountably neglected.

### THE WOMAN COMMITTEEMAN.

King's College Hospital has now decided to admit women to the committee of management, and the first to be elected are Lady Hambleden, chairman of the Ladies' Association, and the Hon. Mrs. Anthony Henley. We suppose that few people of experience would now deny that mixed committees generally work extremely well, because perhaps men and women are mutual critics of each other, and thus can interpose to stop discursive debates with less offence and more effect than is possible when a committee is confined to one sex only.

### NEW BUILDING FOR WEST CUMBERLAND INFIRMARY?

A remarkable announcement was made to a special meeting of the Governors of the Whitehaven and West Cumberland Infirmary by their new chairman, Colonel J. A. Jackson. A building scheme to spend £12,000, of which about half has been raised, had been proposed, but the chairman said that he had received a letter from some anonymous donors, the full terms of which would be announced later, urging the postponement of the building scheme. In effect the donors said that a considerable sum would be available to prevent expenditure on the present building. The chairman implied that the money would be available for spending elsewhere, and said that in a few weeks the negotiations would be complete. The Governors agreed to adjourn the meeting.

### A SECOND-HAND BOOK FUND.

A letter signed Clement Belcher in the *Birmingham Post* contains a proposal for raising funds for hospitals by the sale at a central store of second-hand books given freely by supporters of hospitals. The writer points out that many people have dozens of books for which they have no further use, and himself offers a hundred volumes which he would be pleased "to give to a fund for the hospitals, which would sell at one or two shillings each, and should be willing to replace them by buying books of a similar value." He suggests that a large exchange for the catalogue of these books should be organised. There is something in the suggestion, because such a casual collection from all and sundry might contain books of unsuspected value. There are many people not interested in literature who possess volumes of interest which never come into the book market. But the proposer's suggestion would have to be widely followed if a large sum is to be raised from small sums.

### A SPLENDID GIFT TO NOTTINGHAM.

In commemoration of his seventieth birthday and also "to assist those sufferers who have been less fortunate than myself," Sir Jesse Boot has subscribed £50,000 to the Nottingham General Hospital. He has stipulated that the sum shall be placed to capital account, and invested for the time being in Nottingham Corporation Six per

Cent. Housing Bonds. There was a danger of the two convalescent homes attached to the hospital closing for lack of funds, and the interest of Sir Jesse's gift is to be applied to this special object, thus freeing the hands of the Committee of the General Hospital in respect of their main work. The connection between the housing bond campaign and charitable institutions is not new—for instance, at Richmond subscribers have made valuable gifts in this form. It was only a few days ago that Sir Jesse Boot announced his intention to give £50,000 in furtherance of the East Midlands University Scheme.

### MEDICAL WOMEN AND MARRIAGE.

The 1920 report of the London (Royal Free Hospital) School of Medicine for Women contains a list of approximately 750 registered medical women who were formerly students of the school. Of these we notice that thirty-seven were married before becoming qualified and 197 have entered upon matrimony after. Many of the medical women in fact nearly all of them, marry medical men, and presumably continue to exercise their profession, for only twenty-nine of the whole list, married and unmarried, have retired from practice. Most of the women on the list hold appointments under public authorities and in voluntary and other hospitals and infirmaries. A scheme for developing medical education of women on the lines of the clinical unit has been carefully thought out, and an effort is being made to collect £500,000 for the enlargement of the Royal Free Hospital and the Medical School. The weekly board of the hospital have appointed Miss May Beeman, O.B.E., so well-known in connection with the Alexandra Day collections, to act as appeal secretary.

### ST. GEORGE'S HOSPITAL.

Those who have recently passed across the large expanse where converging roads meet at Hyde Park Corner, incidentally visualising the proposed war memorial in the Egyptian style, have remarked that, although that much-debated structure has faded like the insubstantial pageant described by Prospero, St. George's Hospital has every appearance of entering upon another two hundred years of useful work on the finest site in London. The painters' cradles hang over the roof of the great building and already the stucco work of the upper floors has been reduced from Metropolitan grime to an almost beautiful grey. The exigencies of the appeal, alas! have caused the fine portico to be obscured. In our issue of April 19, 1919, we published an illustration showing the elevation of the proposed new building.

### DELAYED RECOGNITION OF SIGNAL SERVICE.

Dr. John F. Williams, of Southwark, has been awarded the medal of the Order of the British Empire "for courage and devotion to duty on the occasion of an air raid in rescuing persons who had been buried in the cellar of a burning building." Dr. Williams is medical officer of detachment 278, Southwark Division, of the British Red Cross, which did splendid work when South London was attacked in the air raids. The bald statement of the award recalls one of the most disastrous of the air raids. A bomb from a Zeppelin crashed into Albany Road, Camberwell, a thickly-populated district. It could not have fallen at a better place for the invaders, for it descended at the corner where four streets of dwelling-houses are situated. It crashed through the surgery of Dr. Whitlaw at the corner and an adjoining fish shop, killing ten people who had taken shelter in the basements. Apart from the deaths and injuries the scene of devastation was appalling, and over 300 people were rendered homeless. Dr. Williams and his helpers had a hard task in October 1916, but their courage and devotion to duty did much to alleviate the havoc. A visit to the stricken district the day after the bomb fell showed what damage and injuries could be done by a solitary bomb. It was undoubtedly the worst disaster that befell South London, and the tardy recognition of the leader of the gallant band of rescuers should be all the more appreciated.

### HOSPITAL BENEFITS BY CRICKET WEEK.

Canterbury Cricket Week is far-famed, and during the seven days which commenced last Saturday the ancient city once again gave itself up to the enjoyment of this event. A number of entertainments were arranged for the gratification of the many thousands of visitors, and it is pleasing to know that this year most of these entertainments are for the benefit of the Kent and Canterbury Hospital. In addition to a carnival through the streets, when collections were made for the institution, a series of pastoral plays was given by [illegible] leading Shakespearean company in the historic setting of St. Augustine's Abbey. There was also a military tattoo, an open-air concert, and a boxing tournament, the profits from all of which go to the hospital. A leading and active part in these arrangements was taken by the Mayor of Canterbury, Councillor James, a Vice-President of the hospital. The various entertainments mentioned were supplemented by special collections for the hospital at the Cathedral on Sunday, August 1.

### THIS WEEK'S DRUG MARKET.

Very little business has been done since our last report, and the market has only just recovered from the effect of the holiday. Although quotations, generally speaking, move downwards but slowly, there are here and there offers at substantially below the nominal market prices, and it would not [illegible] surprising if such offers became more numerous, [illegible] holders must in some instances be anxious to turn their goods into cash. Some of the synthetic drugs, as, for instance, phenacetin and phenazone, have been offered at comparatively low rates, and potassium bromide, which is getting more plentiful, is available at considerably reduced prices. Cocaine is in more plentiful supply, and is somewhat cheaper. In consequence of the fall in the price [illegible] citric acid, makers of citrates have reduced their quotations. Cream of tartar is also cheaper. More business is being done in cod-liver oil. Lemon [illegible] has moved downwards in price.

